# *Toxoplasma gondii* excretory/secretory antigens (TgESAs) suppress pro-inflammatory cytokine secretion by inhibiting TLR-induced NF-κB activation in LPS-stimulated murine macrophages

**DOI:** 10.18632/oncotarget.19362

**Published:** 2017-07-18

**Authors:** Shuai Wang, Zhenchao Zhang, Yujian Wang, Javaid Ali Gadahi, Qing Xie, Lixin Xu, Ruofeng Yan, Xiaokai Song, Xiangrui Li

**Affiliations:** ^1^ College of Veterinary Medicine, Nanjing Agricultural University, Nanjing, Jiangsu, PR China; ^2^ School of Basic Medical Sciences, Xinxiang Medical University, Xinxiang, Henan, PR China

**Keywords:** toxoplasma gondii, excretory/secretory antigens, macrophage, cellular function, immunomodulation, Immunology and Microbiology Section, Immune response, Immunity

## Abstract

Excretory/secretory antigens (ESAs) produced by *Toxoplasma gondii* enable this parasite to invade and survive within the host cells through immunomodulation. In this study, the modulating effects of *T. gondii* excretory/secretory antigens (TgESAs) on the Ana-1 murine macrophage cell line were evaluated. Ana-1 cells were incubated with several concentrations of TgESAs, and the resulting effects on cellular viability, phagocytotic ability, and apoptosis induction were determined. Pro-inflammatory and anti-inflammatory cytokine secretion, nitric oxide production, toll-like receptor expression, and nuclear translocation of NF-κB were all measured after incubation with TgESAs. After TgESAs treatment, the proliferation and phagocytosis ability of Ana-1 cells decreased, and apoptosis was induced in a dose dependent manner. Analysis of Ana-1 cell culture supernatants showed that TgESAs not only upregulated secretion of anti-inflammatory cytokines (interleukin-10 and transforming growth factor-β1), they also inhibited secretion of pro-inflammatory cytokines (tumor necrosis factor-α and interleukin-1β). Additionally, TgESAs inhibited nitric oxide production, toll-like receptor (TLR) 2 and 4 activation, and the nuclear translocation of NF-κB in lipopolysaccharide-stimulated Ana-1 macrophages. These results suggest TgESAs inhibit the functional activity of Ana-1 murine macrophages by inhibiting TLR-induced NF-κB activation, which suppresses pro-inflammatory cytokine secretion.

## INTRODUCTION

Toxoplasmosis is caused by *Toxoplasma gondii,* which is widespread in humans and animals and is an opportunistic pathogen infecting patients that are immunocompromised [1]. During *T. gondii* infection, the parasite releases a number of molecules termed *T. gondii* excretory/secretory antigens (TgESAs) into its surrounding environment, which enables the organism invade and survive within the host cells through immunomodulation [2]. These TgESAs might be one of the first targets of the host’s immune system, and could be a valuable candidate for toxoplasmosis diagnosis and useful for the development of immunization strategies [3-6].

TgESAs from both the virulent and less virulent *T. gondii* strains display a chemokine-like activity, which leads to a dysfunctional dendritic cell-mediated immune response [7]. In murine bone marrow-derived macrophages, TgESAs not only inhibited the up-regulation of major histocompatibility complex (MHC) class II molecules, they also inhibited the release of tumor necrosis factor-α (TNF-α), a pro-inflammatory cytokine [8].

Macrophages resist toxoplasmosis by limiting parasitic replication and releasing cytokines that inhibit *T. gondii* infection [9-11]. Macrophages, a target for immunomodulation by the *T. gondii*, play a critical role in initiating and modulating the host immune response to *T. gondii* infection. In the present study, we examined the effect of TgESAs on modulating Ana-1 macrophage activities, which laied the foundation for further understanding of the effective immune-evasion mechanism used by *T. gondii*.

## RESULTS

### TgESAs decreased Ana-1 cell viability, induced apoptosis

**Figure 1:**
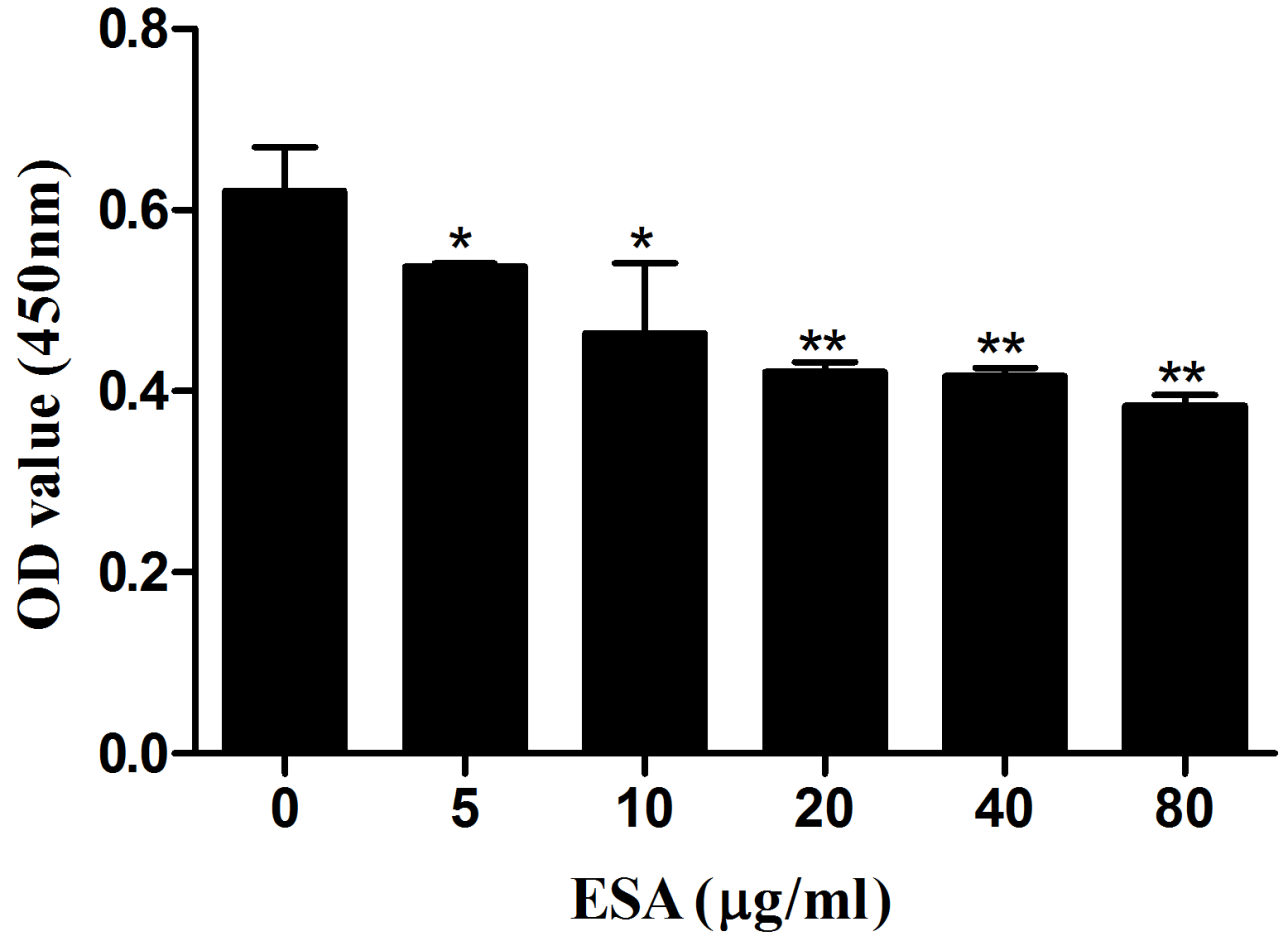
Effect of TgESAs on Ana-1 cell viability Values are mean ± standard deviation of three independent experiments. *P < 0.05 and **P < 0.01 compared with untreated group (0 μg/ml).

**Figure 2:**
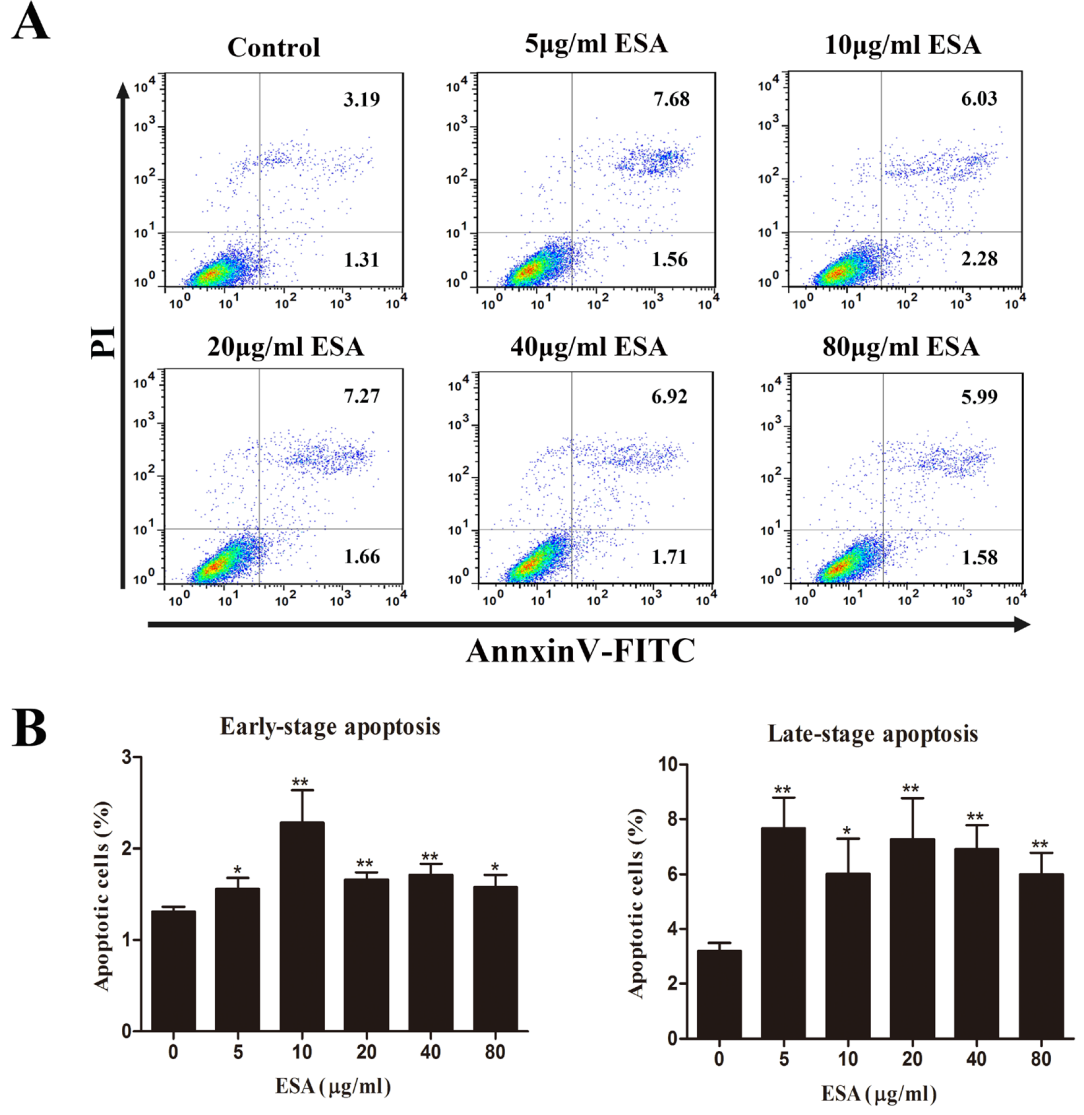
TgESAs induce Ana-1 cell apoptosis Ana-1 cells were treated for 48 h with different concentrations (0, 5, 10, 20, 40, 80 μg/mL) of TgESAs. (A) The flow cytometry data showed one representative dual staining result. (B) Early- stage (%) and late-stage (%) apoptotic cell death were detected by staining cells with Annexin V-FITC and PI, and analyzing by flow cytometry. *P < 0.05 and **P < 0.01 compared with untreated group (0 μg/ml).

Ana-1 cells were exposed to different concentrations (0, 5, 10, 20, 40, 80 μg/mL) of TgESAs for 48 h, and the resulting cell viability was assessed. CCK-8 assay results showed that TgESAs significantly decreased Ana-1 cell viability (Figure 1). Decreased cell viability could be caused by the induction of apoptosis, so we measured the amount of early- and late- stage apoptotic cells. Both early-stage and late-stage apoptosis were induced by different concentrations of TgESAs at 48 h (Figure 2A, 2B).

### Capacity of phagocytosis

**Figure 3:**
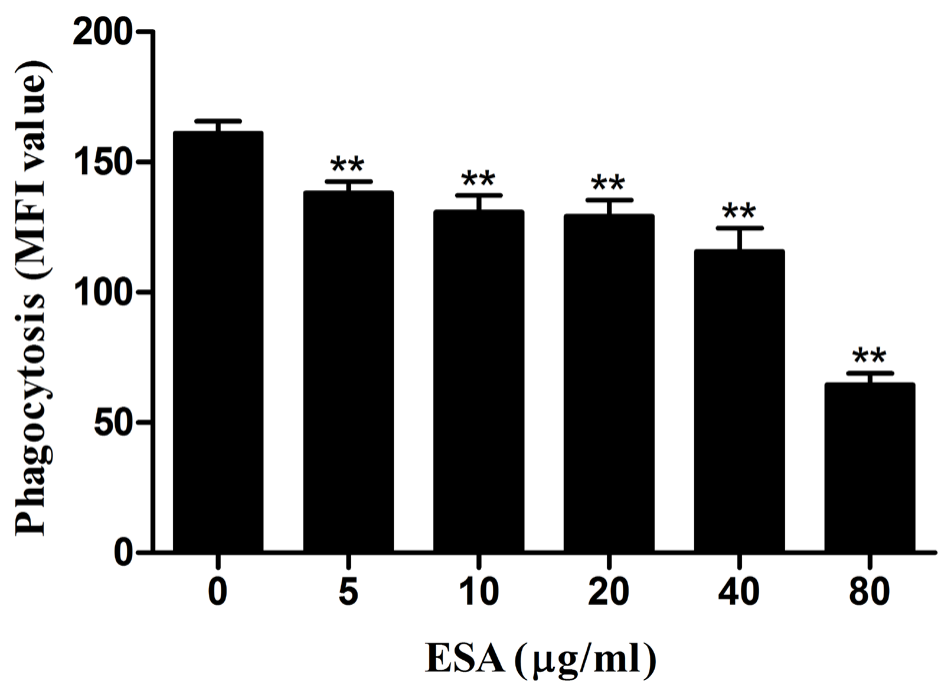
Effect of TgESAs on the phagocytosis of Ana-1 cells by flow cytometry Ana-1 cells were treated for 48 h with different concentrations (0, 5, 10, 20, 40, 80 μg/mL) of TgESAs. Group histograms showing the median fluorescence intensity (MFI) values. Values are mean ± standard deviation of three independent experiments. *P < 0.05 and **P < 0.01 compared with untreated group (0 μg/ml).

Phagocytic capacity of Ana-1 cells was examined after a 48-hour TgESAs treatment. As TgESAs concentration increased, Ana-1 cells’ ability to uptake FITC-dextran decreased significantly (**P < 0.01, Figure 3).

### TgESAs suppressed pro-inflammatory cytokine production

**Figure 4:**
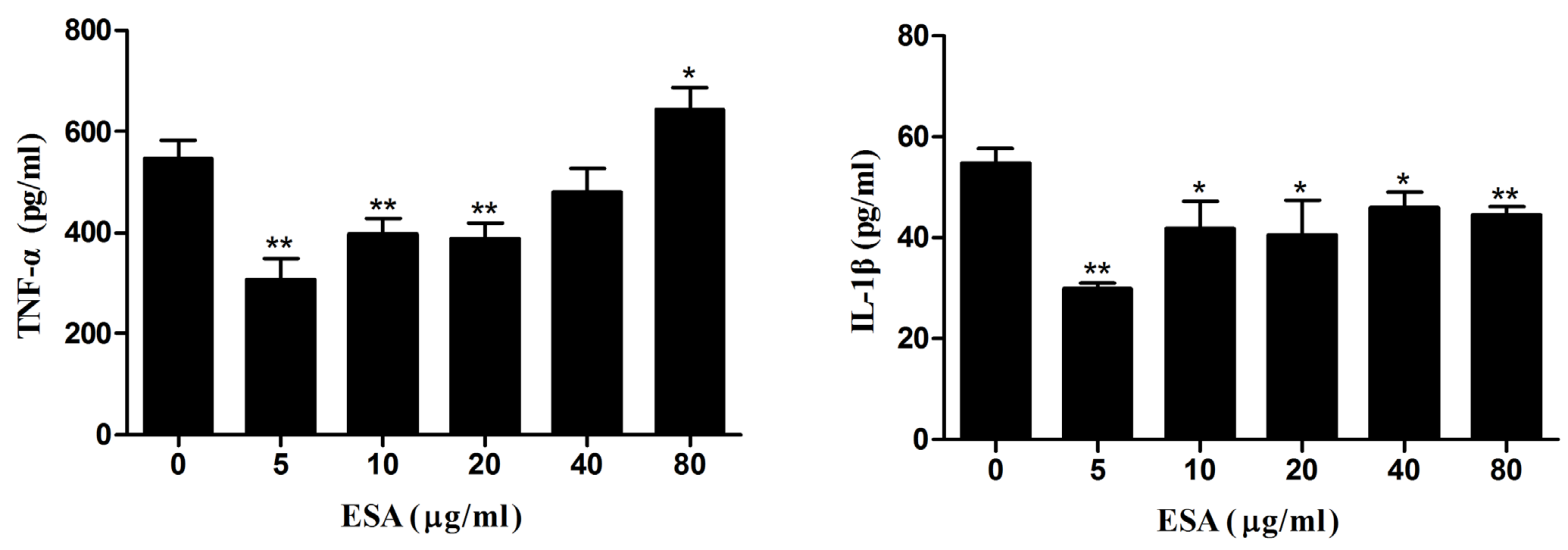
Production of pro-inflammatory cytokines in Ana-1 cells Ana-1 cells were treated for 48 h with different concentrations (0, 5, 10, 20, 40, 80 μg/mL) of TgESAs before LPS stimulation at 100 ng/ml for 12 h. Values are mean ± standard deviation of three independent experiments. *P < 0.05 and **P < 0.01 compared with untreated group (0 μg/ml TgESAs plus LPS).

Pre-treatment with TgESAs of LPS-stimulated Ana-1 macrophages significantly reduced TNF-α release and IL-1β secretion (*P < 0.05, **P < 0.01; Figure 4), with 5 µg/mL ESA showing the highest inhibitory activity.

### TgESAs increased anti-inflammatory cytokine production

**Figure 5:**
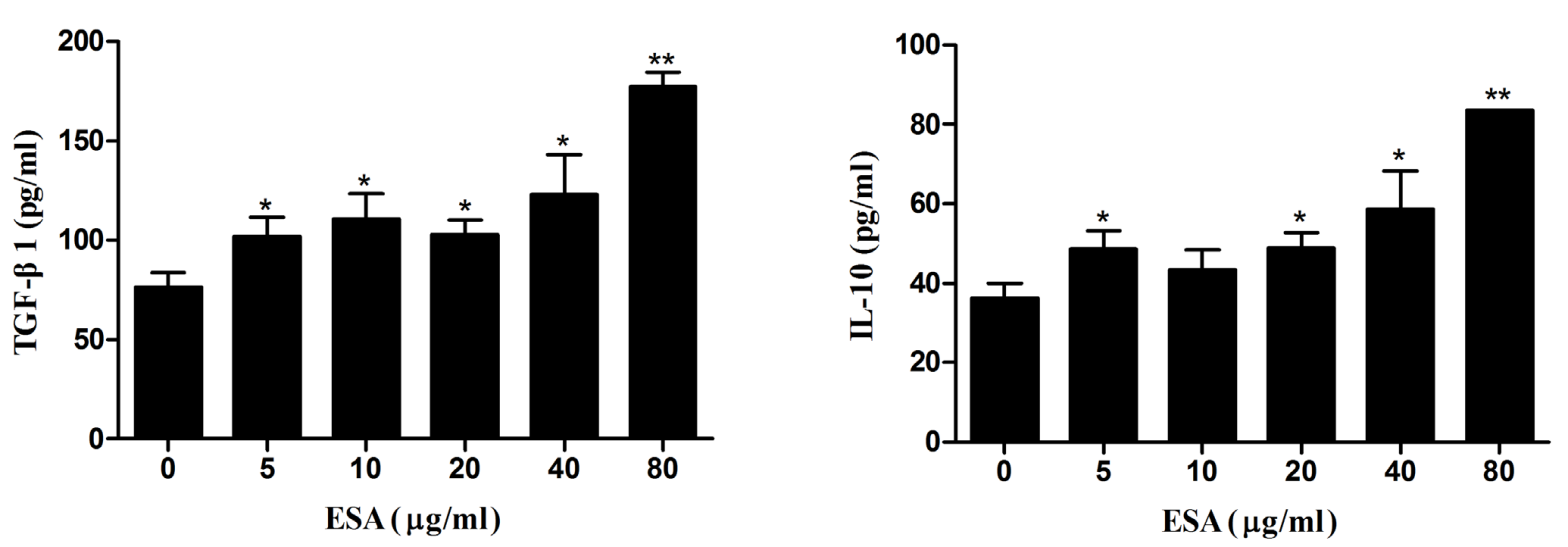
Production of anti-inflammatory cytokines in Ana-1 macrophages Ana-1 cells were treated for 48 h with different concentrations (0, 5, 10, 20, 40, 80 μg/mL) of TgESAs. Values are mean ± standard deviation of three independent experiments. *P < 0.05 and **P < 0.01 compared with untreated group (0 μg/ml).

TgESAs-induced anti-inflammatory cytokine secretion was examined in Ana-1 macrophages using ELISA. Compared with control, TgESAs significantly increased IL-10 and TGF-β1 secretion (*P < 0.05, **P < 0.01) in Ana-1 cells according to dose (Figure 5).

### NO production

**Figure 6:**
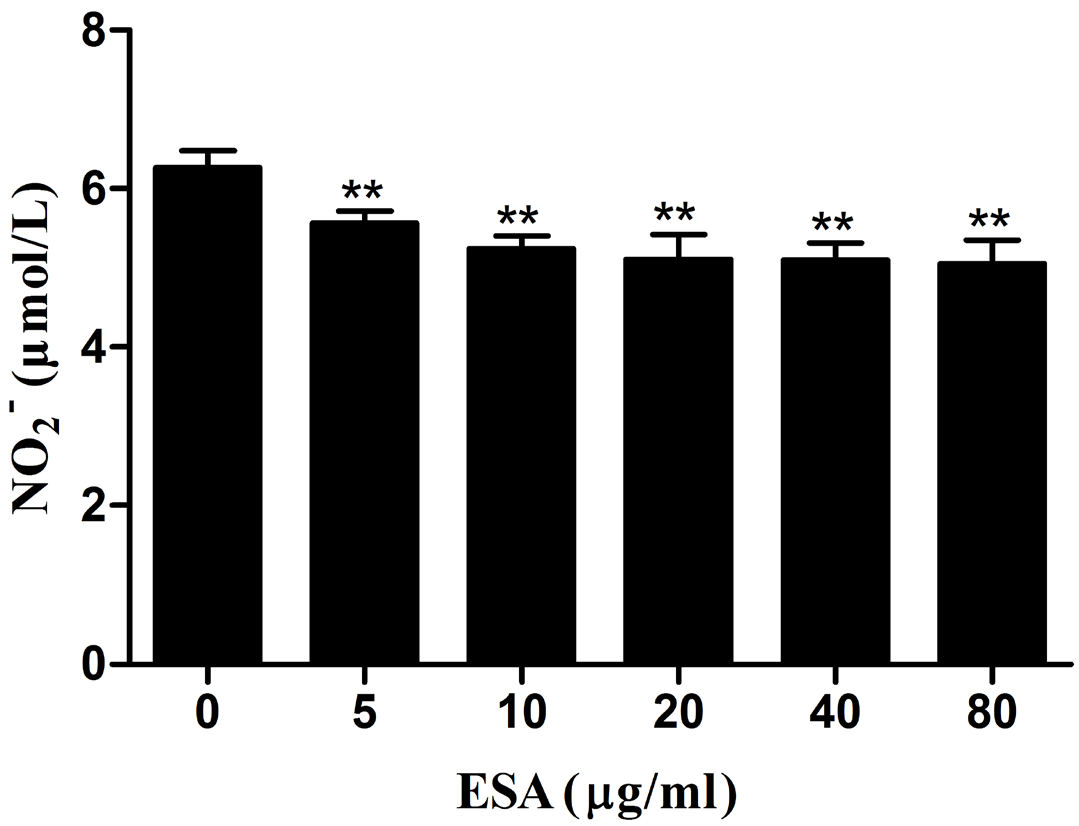
Effect of TgESAs on the NO production of Ana-1 cells Ana-1 cells were treated for 48 h with different concentrations (0, 5, 10, 20, 40, 80 μg/mL) of TgESAs before LPS stimulation at 100 ng/ml for 12 h. Values are mean ± standard deviation of three independent experiments. *P < 0.05 and **P < 0.01 compared with untreated group (0 μg/ml TgESAs plus LPS).

The effects of TgESAs on NO production in LPS-stimulated Ana-1 cells were examined. After TgESAs treatment, culture supernatants of Ana-1 cells showed decreased nitrate concentrations (**P < 0.01, Figure 6).

### TgESAs inhibited NF-κB activation in LPS-stimulated Ana-1 macrophages

**Figure 7:**
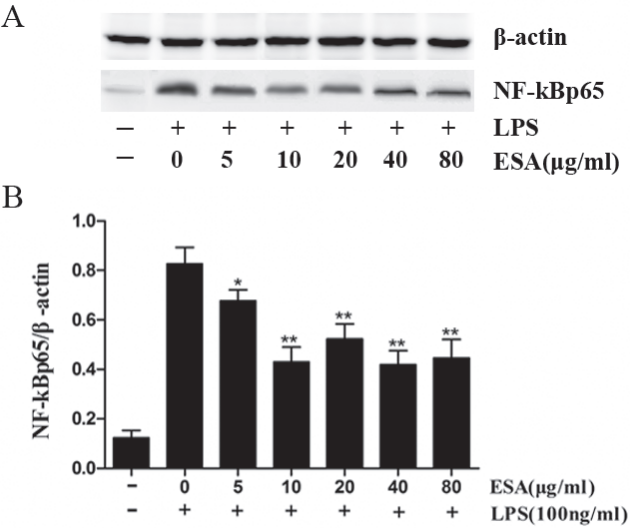
Effect of TgESAs on the activation of NF-kB, detected by Western blotting (A) The nuclear NF-kB protein expression of Ana-1 macrophage cells that were treated with 0, 5, 10, 20, 40 or 80 μg/ml ESAs from *T. gondii* for 48 h before LPS stimulation at 100 ng/ml for 1 h. (B) The results were analyzed by Quantity One software and stated in NF-kB *vs* β-actin. Values are mean ± standard deviation of three independent experiments. *P < 0.05 and **P < 0.01 compared with control group (0 μg/ml TgESAs plus LPS).

NF-κB levels were very low in the nucleus of the no treatment control (Figure 7). However, high nuclear NF-κB levels were observed after LPS treatment for 1 h. A 48h pre-treatment of Ana-1 macrophages with TgESAs significantly inhibited the LPS-induced NF-κB activation.

### TLR2 and TLR4 expressions

**Figure 8:**
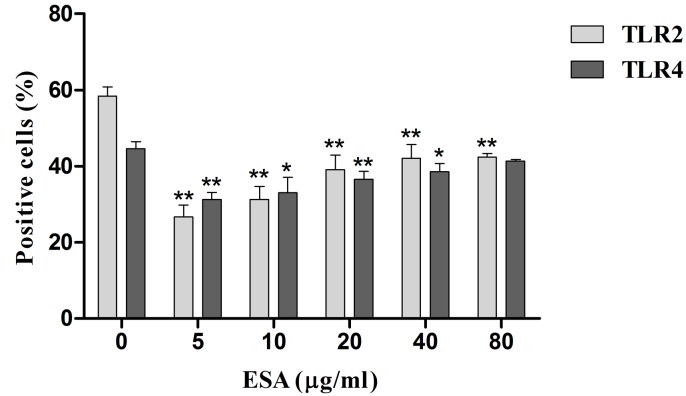
TLR2 and TLR4 expression in Ana-1 cells by flow cytometry Ana-1 cells were treated for 48 h with different concentrations (0, 5, 10, 20, 40, 80 μg/mL) of TgESAs before LPS stimulation at 100 ng/ml for 1 h. Values are mean ± standard deviation of three independent experiments. *P < 0.05 and **P < 0.01 compared with untreated group (0 μg/ml TgESAs plus LPS).

We assayed the percentage of cells positive for Toll-like receptor (TLR) 2 or 4. The 48h TgESAs pre-treatment suppressed TLR2 and TLR4 expression of LPS-stimulated Ana-1 cells (*P < 0.05, **P < 0.01; Figure 8).

## DISCUSSION

In the present study, TgESAs decreased Ana-1 cell viability and induced both early- and late-stage apoptosis. Inducing apoptosis could actually be the cause of the decreased cell viability. In agreement with our findings, the culture supernatant of *T. gondii* inhibited the proliferation and caused apoptosis of human gastric cancer BGC-823 cells, which might be related with up-regulating p53 expression and down-regulating Bcl-2 expression [12]. The exact mechanism of TgESAs-induced macrophage apoptosis needed further research.

Effective macrophages engulf and kill pathogens [13], so we studied the phagocytic capacity of Ana-1 cells in the present study. After TgESAs treatment, phagocytic capacity of Ana-1 macrophages decreased, which was favorable to parasite survival within macrophages.

Activated macrophages produce nitric oxide (NO), that is important to control the multiplication of *T. gondii* [14]. However in murine macrophages, *T. gondii* infection partially inhibits NO production due to iNOS degradation [14]. In the present study, TgESAs inhibited the NO production of murine macrophages. The reduced NO production weakens the intracellular killing effect of macrophages on *T. gondii*, which would provide an effective evasion mechanism.

Pro-inflammatory cytokines such as IL-1β, IL-18, IL-12, IFN-γ, and TNF-α are critical for the host resistance against *T. gondii* [15, 16], while anti-inflammatory cytokines like IL-10 and TGF-β inhibit the host resistance *via* suppressing the secretion of pro-inflammatory cytokines [17-20]. We found that after TgESAs incubation, Ana-1 cells secreted less TNF-α and IL-1β, but more IL-10 and TGF-β1. This suggests that TgESAs inhibit pro-inflammatory and stimulate anti-inflammatory cytokine expression in *T. gondii*-infected macrophages. Both of these effects establish an anti-inflammatory microenvironment that is favorable to parasitic replication.

In this study, we found that treating Ana-1 macrophages with TgESAs significantly inhibited the LPS-induced nuclear translocation of NF-κB p65. By inhibiting the NF-kB transcription factor, *T. gondii* tachyzoites suppress TNF-α and IL-12 secretion, which are pro-inflammatory cytokines [17].

TLR2 and TLR4 promote the recognition and stimulation of immune responses against *T. gondii* [21, 22]. In this study, TgESAs suppressed both TLR2 and TLR4 expression in Ana-1 cells. TLR4 can use both MYD88- and TRIF-dependent pathways to active the downstream pro-inflammatory transcription factor NF-κB [23]. TLR4 down-regulation inhibits NF-κB signaling, which is followed by the reduction of pro-inflammatory cytokines TNF-α and IL-1β. We demonstrated that TgESAs suppress pro-inflammatory cytokine secretion by inhibiting TLR-induced NF-κB activation.

In conclusion, TgESAs from the virulent RH strain (Type I) inhibited the functional activity of murine macrophage Ana-1 cells. These data reinforced the understanding of strategies used by *T. gondii* to evade anti-parasitic mechanisms of the host cell. *T. gondii* strains are highly diverse but only a few lineages (Type I, II and III) are widely spread [24]. Hence, it might need further study whether TgESAs from the less virulent strain (Type II) or non-virulent strains (Type III) exhibit the same inhibitory effects on functions of murine macrophages. Additionally, the discrete immunomodulators exhibiting the inhibitory effects on host macrophages should also be identified in future research.

## MATERIALS AND METHODS

### Ethics statement

This study was approved by the Ethical Committee of Animal Experiments of the College of Veterinary Medicine, Nanjing Agricultural University, China. All experimental protocols were approved by the Science and Technology Agency of Jiangsu Province. The approval ID is SYXK (SU) 2010-0005.

### *T. gondii* strain

*T. gondii* RH tachyzoites were grown and maintained in BALB/c mice by intraperitoneal inoculation. Three to four days after infection, the peritoneal fluids from infected mice were collected in PBS. Tachyzoites were harvested by passage twice through a 27-gauge needle followed by filtration through a membrane with 5 μm pores (Millipore Corp., Bedford, MA, USA) to remove host cell debris. Parasites were washed twice, counted, and suspended in fetal bovine serum-free RPMI 1640 medium for preparing excretory/secretory antigens.

### Preparation of *T. gondii* excretory/secretory antigens (TgESAs)

TgESAs were obtained according to a previously-described method [4] with minor modifications. Briefly, *T. gondii* tachyzoites (approximately 10^8^ tachyzoites/ml) were resuspended in RPMI 1640 containing 100 IU/ml penicillin and 100 μg/ml streptomycin. Next, they were transferred to sterile 15 ml conical centrifuge tubes and incubated at 37 °C for 3 h with gentle shaking under sterile conditions. After incubation, tubes were centrifuged at 1000×g for 10 min, and their supernatants were collected. The supernatants referred to as TgESAs were concentrated by Millipore ultrafiltration tube (MWCO 3,000, Millipore Corp., Bedford, MA, USA). TgESAs were treated with AffinityPak Detoxi-Gel Endotoxin Removing Gel (Thermo Fisher Scientific, Waltham, MA, USA) to achieve an endotoxin level < 0.1 EU/mg and filtered through a 0.22 μm millipore membrane filter (Millipore Corp., Bedford, MA, USA), and stored at -80^o^C until use. The protein concentration of TgESAs was determined with a BCA protein assay kit (Bio-Rad, Hercules, CA, USA).

### Cell culture

The Ana-1 mouse macrophage cell line (Institute of Cell Biology, Chinese Academy Sciences, Shanghai, China), were cultured in RPMI 1640 medium containing 10% heat-inactivated fetal bovine serum (FBS), 100 U/ml penicillin, and 100 mg/ml streptomycin at 37°C in a 5% CO_2_ atmosphere.

### Cell viability assay

A CCK-8 assay was performed to measure the effect of TgESAs on cell viability of Ana-1 cells. Ana-1 cells were cultured in 96-well plates at a density of 2×10^5^ cells/ml in the presence of different doses (0, 5, 10, 20, 40, 80 μg/mL) of TgESAs at 37°C in 5% CO_2_ incubator for 48 h. After incubation, 10 μL of CCK-8 solutions were added to each well of the plate for additional 2 h of incubation. The optical density of each well was measured at 450 nm using a microplate reader (Molecular Devices Co, Sunnyvale, California, USA). The data are expressed as the mean ± the standard deviation of the mean (SD) for at least three independent experiments.

### Macrophage treatments

Three treatments were performed on the Ana-1 murine macrophage cell line: (a) Cells were treated for 48 h with different concentrations (0, 5, 10, 20, 40, 80 μg/mL) of TgESAs. (b) Cells were treated for 48 h with different concentrations (0, 5, 10, 20, 40, 80 μg/mL) of TgESAs before stimulation with 100 ng/ml lipopolysaccharides (LPS) for 1 h. (c) Cells were treated for 48 h with different concentrations (0, 5, 10, 20, 40, 80 μg/mL) of TgESAs before stimulation with 100 ng/ml LPS for 12 h.

### Determining nitric oxide (NO) concentration in cell supernatants

The Ana-1 cells were treated with different concentrations (0, 5, 10, 20, 40, 80 μg/mL) of TgESAs for 48 h, followed by treatment with LPS (100 ng/ml) for an additional 12 h. Cell supernatants were collected and analyzed for nitrite (NO_2_^-^) accumulation as an indicator of NO production using a NO assay kit (Beyotime Institute of Biotechnology, Haimen, Jiangsu, China) according to the manufacturer’s protocol. Briefly, a standard curve was prepared with standard nitrite solutions in DMEM medium. The standard solutions or cell supernatants were reacted with nitrate reductase for 30 min in a 96-well plate, and then Griess reagent I and Griess reagent II were added. After a 10 min incubation at room temperature, the absorbance at 540 nm was read in a microplate reader. The samples were assayed in triplicate.

### Cytokine ELISA

The levels of TNF-α, IL-1β, IL-10, and TGF-β1 in treated macrophage cell supernatants were determined using commercially available ELISA kits according to the manufacturer’s instructions (Boster Systems, Wuhan, China). The cytokine concentrations were determined by reference to standard curves constructed with known amounts of mouse recombinant TNF-α, IL-1β, IL-10, and TGF-β1. The analysis was performed with data from three independent experiments.

### FITC-dextran internalization

To confirm the effect of TgESAs on the phagocytotic ability of Ana-1 cells, the FITC-dextran internalization of cells was analyzed by flow cytometry. Cells were collected after treatment with TgESAs for 48 h and incubated with FITC-dextran (1mg/ml in RPMI 1640) for 1 h at 37°C. Cells added with the same amount of FITC-dextran and incubated at 4°C for 1 h were used as the baseline of macrophage phagocytosis. After incubation, wells were washed extensively to remove excess FITC-dextran. The FITC-dextran internalization of cells was analyzed by flow cytometry (BD Biosciences, San Jose, CA, USA) using Cell Quest Software, and median fluorescence intensity (MFI) was calculated.

### Cell apoptosis assay

The induction of apoptosis in Ana-1 cells by TgESAs was quantified by Annexin V-FITC and PI dual staining using an Annexin V-FITC Apoptosis Detection Kit, performed according to the manufacturer’s instructions (Miltenyi Biotec Inc, Auburn, CA, USA).

### TLR2 and TLR4 expressions on Ana-1 cell surface

The Ana-1 cells were treated with different concentrations (0, 5, 10, 20, 40, 80 μg/mL) of TgESAs for 48 h, followed by treatment with LPS (100 ng/ml) for 1 h. Ana-1 cells were harvested and washed with PBS containing 0.5% FBS and 0.05% sodium azide. Then, cells were stained at 4°C for 30 min with FITC-conjugated anti-mouse Toll-like receptor 2 (CD282) or PE-conjugated anti-mouse Toll-like receptor 4 (CD284) antibody. Finally, cells were fixed in PBS containing 1% paraformaldehyde. Expressions of TLR2 and TLR4 of cells were analyzed by flow cytometry (BD Biosciences, San Jose, CA, USA) using Cell Quest Software.

### Preparation of nuclear protein

Nuclear protein was extracted using a nuclear protein extraction kit (Cat No. 2900; Merck Millipore, Billerica, MA, USA) according to manufacturer’s instructions. The protein concentration was determined with a BCA protein assay kit (Bio-Rad, Hercules, CA, USA).

### Determination of nuclear NF-κB by Western blotting

Extracted nuclear protein was denatured in SDS loading buffer and separated by SDS-PAGE (5%-12% acrylamide gradient gels), then transferred onto Amersham Hybond 0.2 PVDF blotting membrane (GE Healthcare Life Sciences, USA). Rabbit anti-NF-κBp65 monoclonal antibody or rabbit anti-β-actin monoclonal antibody (Cell Signaling Technology, Massachusetts, USA) was applied to the membrane for 1 h at 37°C after blocking with 5% nonfat milk. This was followed by incubation with horseradish peroxidase (HRP) conjugated goat anti-rabbit IgG (Cell Signaling Technology, Massachusetts, USA). Finally, the proteins were visualized by a chemiluminescence ECL western blotting analysis system (GE Healthcare, Piscataway, NJ, USA). The protein levels were quantified using ImageJ software (National Institutes of Health, Bethesda, MD, USA), and were normalized to β-actin.
